# Sea level variability in Gulf of Guinea from satellite altimetry

**DOI:** 10.1038/s41598-024-55170-x

**Published:** 2024-02-27

**Authors:** Franck Eitel Kemgang Ghomsi, Roshin P. Raj, Antonio Bonaduce, Issufo Halo, Björn Nyberg, Anny Cazenave, Mathieu Rouault, Ola M. Johannessen

**Affiliations:** 1 Department of Oceanography, University of Cape Town, Cape Town, South Africa; 2 Geodesy Research Laboratory, National Institute of Cartography, P.O. Box 157, Yaoundé, Cameroon; 3 Nansen-Tutu Center for Marine Environmental Research, University of Cape Town, Cape Town, South Africa; 4 Nansen Environmental and Remote Sensing Center and Bjerknes Center for Climate Research, Bergen, Norway; 5 Department of Forestry, Fisheries and the Environment, Oceans & Coasts Research, Cape Town, South Africa; 6 7Analytics, Innovation District Solheimsviken 7c, 5054 Bergen, Norway; 7 Laboratoire d’Etudes en Géophysique et Océanographie Spatiales (LEGOS), 18 Av. E. Belin, 31401 Toulouse Cedex 9, France; 8 Nansen Scientific Society, Bergen, Norway

**Keywords:** Climate sciences, Attribution, Climate-change mitigation, Physical oceanography, Environmental impact

## Abstract

Coastal zones with dense populations, low elevations and/or inadequate adaptive capacity are on the frontline of unprecedented impacts from climate change. The Gulf of Guinea (GoG), stretching from Liberia to Gabon, is in particular vulnerable to coastal flooding caused by local and/or climate-induced sea level rise. In this region, interannual to decadal coastal sea level changes remain poorly understood, mainly due to a lack of tide gauge stations. Here we use nearly three decades (1993–2021) of satellite altimetry data to study the link between the Equatorial Atlantic and coastal GoG sea level variability. The rate of mean sea level rise increased from 3.47 to 3.89 ± 0.10 mm/yr from the Equatorial oceanic domain to the GoG coastal area, with an acceleration of 0.094 ± 0.050 mm/yr^2^. This corresponds to a mean sea level rise of about 8.9 cm over the entire altimetry period, 1993–2021. We focus on the (extreme) warm/cold events that occur in both the GoG during Atlantic Niños, and along the Angola-Namibia coast during Benguela Niños. Both events are driven by remote forcing via equatorial Kelvin waves and local forcing by local winds, freshwater fluxes and currents intensifications. Analysis of altimetry-based sea level, sea surface temperature anomalies, 20 °C isotherm based PIRATA moorings, and the Argo-based steric and thermometric sea level allows us to follow the coastal trapped waves (CTWs) along the GoG, and its link with major events observed along the strong Equatorial Atlantic warmings in 2010, 2012, 2019 and 2021. Both 2019 and 2021 warming have been identified as the warmest event ever reported in this region during the last 40 years. A lag of 1 month is observed between equatorial and West African coastal trapped wave propagation. This observation may help to better anticipate and manage the effects of extreme events on local ecosystems, fisheries, and socio-economic activities along the affected coastlines. In order to enable informed decision-making and guarantee the resilience of coastal communities in the face of climate change, it emphasises the significance of ongoing study in this field.

## Introduction

Coastal regions worldwide face an unprecedented challenge due to climate change-induced sea level rise^1,2^. The Eastern Tropical Atlantic (ETA) African coastlines are particularly vulnerable to sea level rise because of the presence of several low-lying megacities, a higher risk of flooding, and the intrusion of seawater into river systems and groundwater reservoirs. As one of the most densely populated regions in the world, between 108 and 116 million Africans are expected to be at risk from sea level rise by 2030, with ~ 77 million in Eastern Tropical Africa. The situation becomes even more alarming when we consider the potential for internal displacement due to climate change. Under a 1.7 °C global warming scenario, 17–40 million people in sub-Saharan Africa could be forced to flee their homes by 2050. This number may increase to 56–86 million under a 2.5 °C scenario, with over 60% of those affected residing in West Africa. Water stress, reduced crop productivity, and sea level rise are one of the reasons why people will move^3^.

**Figure 1:**
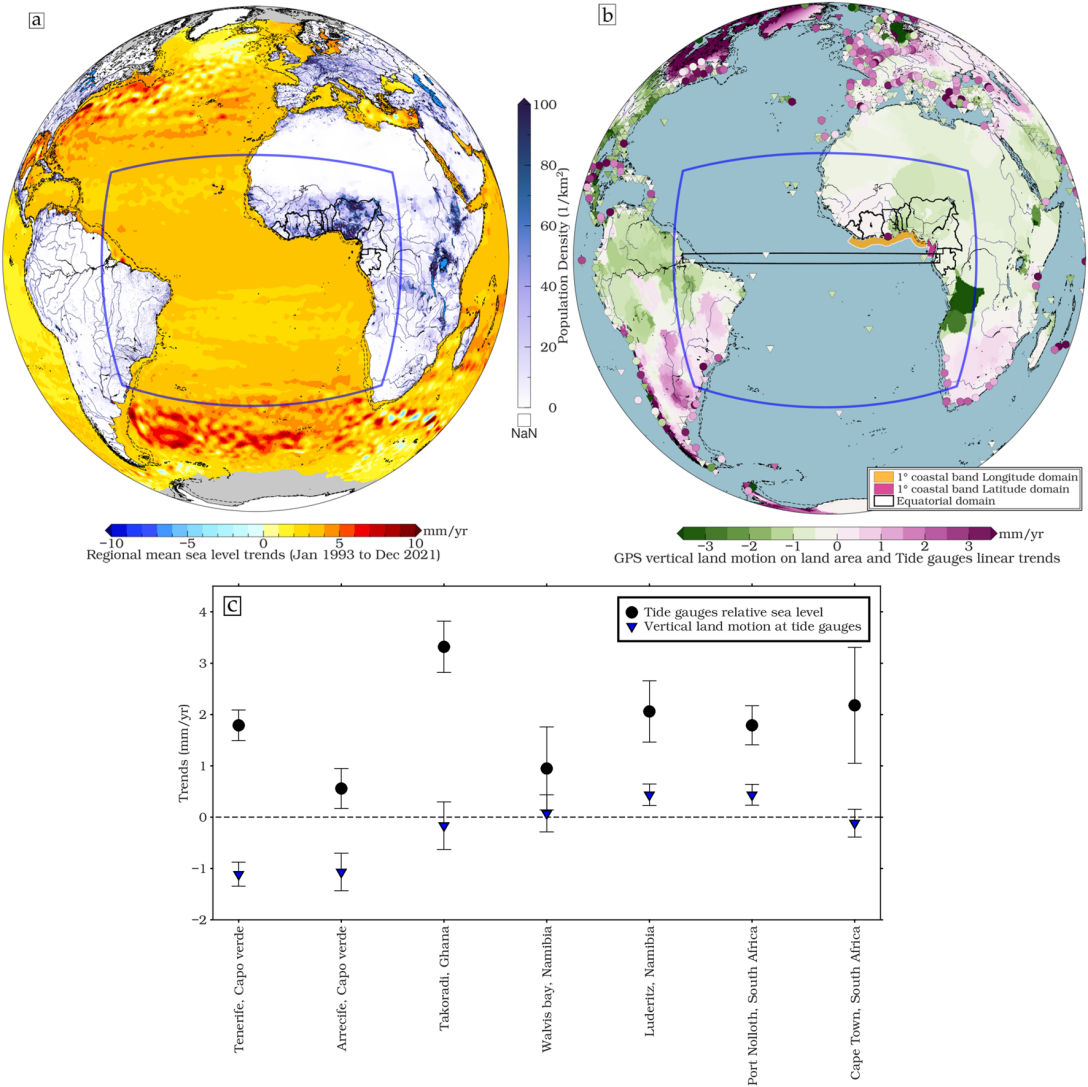
(**a**) Regional sea level trends in mm/year for the period 1993–2021 and population density distribution in 2021 (inhabitants/km^2^) on the continent, based on Gridded Population of the World (GPW, https://sedac.ciesin.columbia.edu/data/collection/gpw-v4) data, highlighting the tropical Atlantic region (blue rectangle, 20°E–52°W and 20°N–30°S). (**b**) GPS imaging shows land regions' vertical rates, with green signifying a downward trend and purple representing an upward trend^11^, with geographical delineations of equatorial (in black, 10°E–50°W and 1°N–1°S) and GoG coastal zones of interest overlaid: Latitudinal domain (0–4°N, 1° wide coastal fringe) in shaded purple and Longitudinal domain (10°E–10°W, 1° wide coastal fringe) in shaded seagreen. (**c**) Vertical land motion rates at tide gauges (inverted blue triangle, http://geodesy.unr.edu/vlm.php) and tide gauge relative sea level (black circle) with 95% confidence intervals along the Atlantic coast of Africa. The tide gauges considered are those closest to existing GNSS stations near the coast. The GoG consists of the countries shown in black contour along the West African coast (from left to right: Liberia, Côte d'Ivoire, Ghana, Togo, Benin, Nigeria, Cameroon, Equatorial Guinea and Gabon), densely populated (about half a billion people live along the coast) and slightly elevated coastline (black dashed line of 200 and 1000 m isobath) is particularly vulnerable to coastal erosion and sea level rise, where sea level trends from January 1993 to December 2021 with global mean sea level retained, derived from satellite altimetry products available through the Copernicus Marine Service, with a focus on the Atlantic Ocean, are shown. The maps have been created using Generic Mapping Tools (GMT), Version 6.5.0 (https://www.generic-mapping-tools.org/).

The Gulf of Guinea (GoG) is a particularly vulnerable area to sea level rise (Fig. 1a). Located in the ETA off the coast of West Africa, it stretches from Cape Palmas (Liberia) at 7°W to Cap López (Gabon), near the equator via the Bight of Biafra (Nigeria and Cameroon) (Fig. 1), the GoG encompasses sandy beaches, estuaries, deltas, lagoons, and river inlets. The region's population, now exceeding 500 million, has quadrupled over the past five decades^4^. Unfortunately, this area is critically affected by sea level rise and frequent extreme flooding, as highlighted by Almar et al.^5^ This vulnerability accentuates the pressing need for in-depth investigations into the factors contributing to these issues, including the intricate dynamics of sea level rise and extreme events in the region. Furthermore, vertical land motion (VLM) from the nearest GNSS (Global Navigation Satellite Systems) measurements at tide gauges of the Permanent Service for Global Mean Sea Level, as illustrated in Fig. 1b and c, reveals significant subsidence rates ranging between 0.2 and 3 mm/yr within the GoG. In contrast, the sea level rise at Takoradi (Ghana), with a record spanning 41 years, measures 3.32 ± 0.5 mm/yr. These findings highlight the compounding effects of land-level changes that increase the region's vulnerability to sea-level rise and reveal the rate of subsidence in the GoG. These observations pose substantial challenges for local coastal communities in the GoG, underscoring the need for effective mitigation of the impacts of sea level change and subsidence in this critical area. Recent studies have highlighted that a relative sea level rise in numerous coastal cities, such as Lagos (Nigeria), is primarily driven by local subsidence, which can exceed absolute sea level rise by significant values. The reported estimates for subsidence in Lagos range from 2 to 87 mm/yr^6^, with the highest rates observed along the coast and in areas where substantial infrastructure has been constructed on landfills. Furthermore, the unprecedented collapse of buildings has also been linked to land subsidence^7–10^. This implies that the relative sea level rise in relation to land subsidence can result in accelerated and intensified coastal inundation, even in cases where the global sea level increase is relatively low. The intricate dynamics of local subsidence, when coupled with regional sea level rise, amplify the risks faced by vulnerable coastal communities in the GoG, requiring urgent attention and strategic mitigation efforts.

The GoG's coastal upwelling and climate-related extreme events are critical in controlling regional fisheries and the resulting social impacts, particularly from sea level rise related to decrease in upwelling. Coastal trapped waves (CTWs) are often used to explain coastal sea level variability^12,13^, as they play an important role in coastal ocean circulation and can also be a cause of saltwater intrusion and shoreline change^14,15^. The seasonal cycle and interannual variability dominate the sea level variability in the GoG^16,17^. On the other hand, the seasonal cycle of the Sea Level Anomalies (SLA) has important features that occur twice a year, suggesting a semi-annual cycle^18–20^. The seasonal signals of Sea Surface Temperature (SST) and SLA highlight the complex dynamics of the ocean system contributing to the variations in these two key ocean variables. Regional interannual SST anomalies have been explained by local and/or remote forcing^21,22^, further linked to the Atlantic Niño mode, commonly reported as the dominant interannual variability mechanism in the Equatorial Atlantic^23–25^. The latter is characterised by an irregular strong cooling (or warming) of the GoG associated with a strengthening (or weakening) of the western equatorial trade winds, culminating in the boreal summer (May–June-July–August, MJJA)^17,24,26,27^. Moving eastwards towards the Niger river plume, coastal cooling weakens in the eastern part of the GoG. This plume is largely responsible for warming the region’s coastal waters and reducing coastal upwelling. The plume inhibits vertical shear of the water and causes a warming of the upwelling zone of up to 1 °C near 2°E by strongly stratifying the water column vertically^28^.

In the Cold Tongue region (3°S to 3°N latitude; 10°W to 20°W longitude), the interannual variability in the eastern equatorial Atlantic peaks in the boreal summer months of June, July, and August, SSTs can increase by up to 1.5 °C above the climatological value during Atlantic Niño events. This has a significant impact on the West African monsoon^29–31^. Extreme events in the GoG often exceed SST anomalies of 2 °C, leading to drastic shocks to fisheries and thus to local populations^32,33^, as well as to regional rainfall in West Africa^29,34,35^.

A rapid rise in easterly wind in the central-western equatorial Atlantic, due to its close connection to the GoG causes a sizable portion of the annual equatorial upwelling forced to occur remotely. Equatorially trapped Kelvin waves (EKWs) are induced by this impulse and move eastward across the Atlantic until they hit the west coast of Africa. A sizable portion of the EKW energy is transported poleward as CTWs reach the eastern boundary along the zonally oriented coasts of both the GoG and Angola-Namibia coast^12^. The warmest Atlantic Niño events of the entire satellite observation era has been recorded in late 2019^36^ and boreal summer 2021^20,37,38^, of which both events were also reported as a Benguela Niño.

Despite the occurrence of these anomalous events, to date, no study has addressed the impacts or potential teleconnections along the GoG. In this study we investigate the GoG sea level variability and trend using satellite altimetry data since 1993 to the present 2021. The paper is structured as follows. First, we show the current variability of altimetry trends in the tropical Atlantic and towards the coasts of the GoG. Then, we perform a large-scale analysis of EKWs, which propagate eastward and then poleward as CTWs upon reaching the West African coast, using SST and SLA data. Finally, the analysis of PIRATA moorings and Argo-based steric and thermometric sea level data from 1993 to 2021 from the equatorial band to the GoG coastal region is presented. At the end, we summarize the main outcomes of this study and present the data sets and methodology used in the study.

## Results

### Regional SST and SLA variability, trends, and changes

**Figure 2:**
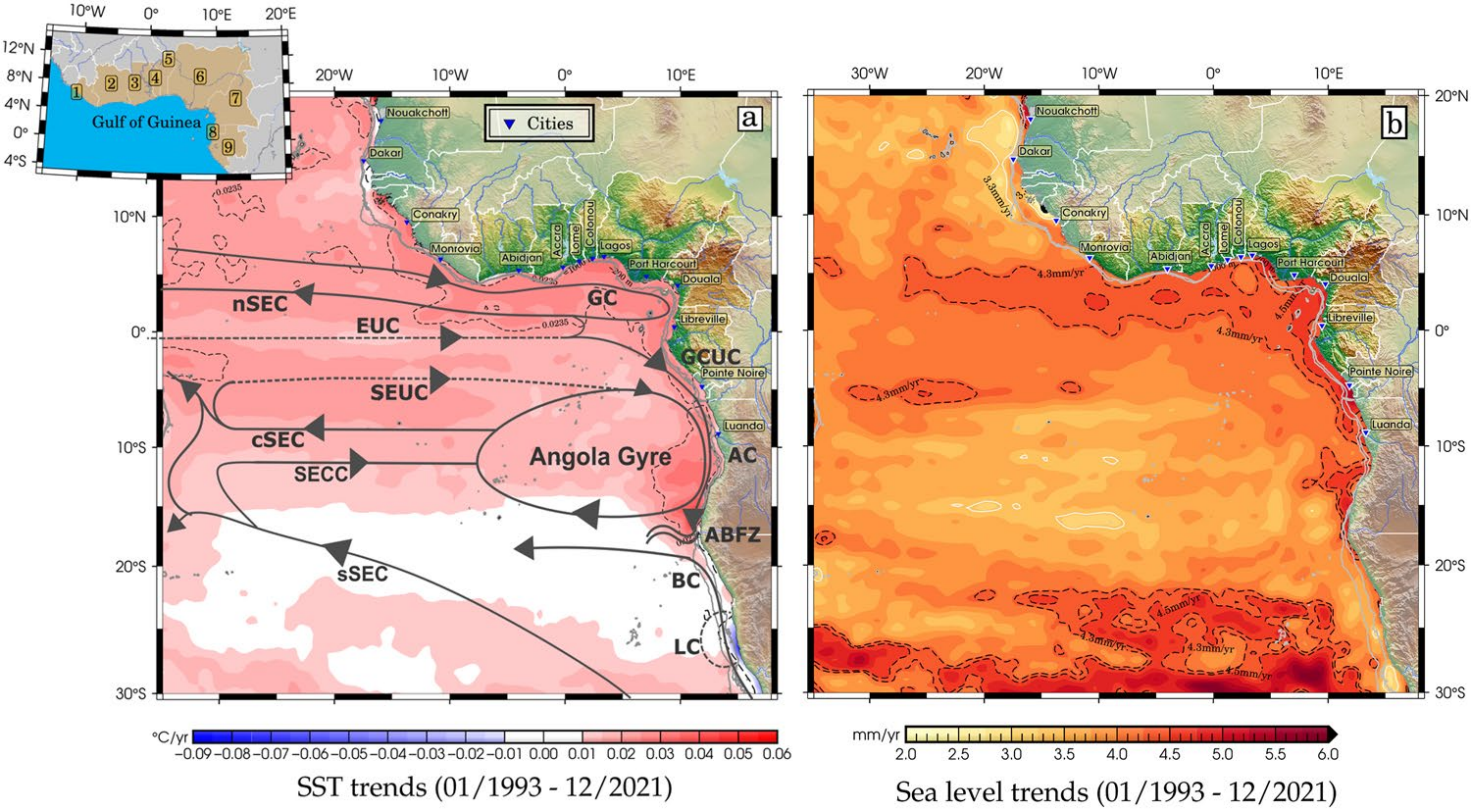
(**a**) Patterns of sea surface temperature (°C/year) with the circulation scheme superimposed and (**b**) sea level (mm/year) trends for the period from January 1993 to December 2021. Dashed black lines represent the common coastal trend maximum and blue lines across Africa represent major river networks. Grey lines represent the 200 and 1000 m isobaths. Surface (solid arrows) and thermocline (dashed arrows) currents^43,44^ shown are the central, northern and southern branches of the Southern Equatorial Current (cSEC, nSEC and sSEC), the Gabon-Congo Undercurrent (GCUC), the Guinea Current (GC), the Equatorial Undercurrent (EUC), the North Equatorial Countercurrent (NECC), the North Equatorial Undercurrent (NEUC), the South Equatorial Countercurrent (SECC), the South Equatorial Undercurrent (SEUC) and the Angola Current (AC). The Angola Gyre, the Angola-Benguela Frontal Zone (ABFZ), the Benguela Current (BC) and the Luderitz Cell (LC) are also shown. The inset shows the countries (with the corresponding cities labelled on the main figures) located in the GoG region and labelled from right to left as follows 1—Liberia (Monrovia); 2—Côte d'Ivoire (Abidjan); 3—Ghana (Accra); 4—Togo (Lomé); 5—Benin (Cotonou); 6—Nigeria (Lagos, Port Harcourt); 7—Cameroon (Douala); 8—Equatorial Guinea; 9—Gabon (Libreville). These maps were created using Generic Mapping Tools (GMT), Version 6.5.0 (https://www.generic-mapping-tools.org/), and the current vectors in Fig. 2.a were designed using Inkscape, Version 1.3 (https://www.inkscape.org/).

Over the last three decades, the ETA has shown significant warming. The spatial patterns of the linear trends in SST and SLA for the period 1993–2021 are shown in Fig. 2a and b. Almost the whole region shows a strong positive trend over this period, but the strengthening is much more pronounced north of the equator near the coast of the GoG. On a decadal timescale, we are interested in how the SST and SLA patterns are evolving and how both variables are connected. Tropical Atlantic displays an overall positive trend in SST (by > 0.0235 ± 0.0051 °C/yr) and SLA (by > 4.3 ± 0.1 mm/yr) from January 1993 to December 2021, mainly in the coastal regions from Conakry to Luanda (see Fig. 2b). The GoG has experienced significant warming, particularly throughout the summer, which intensified from October to April^39^. This is consistent with the findings of Varela et al.^40^, who showed that coastal SSTs are warming faster than the open ocean SST. These significant trends, which extend towards the southern and northeast coastal regions, are consistent with the spatial variability of the SST trend (Fig. 2a), highlighting the likelihood that sea level signals can spread across the ETA coastal domain at both interannual and decadal scales as Kelvin waves trapped at the coast^19,41,42^.

**Figure 3:**
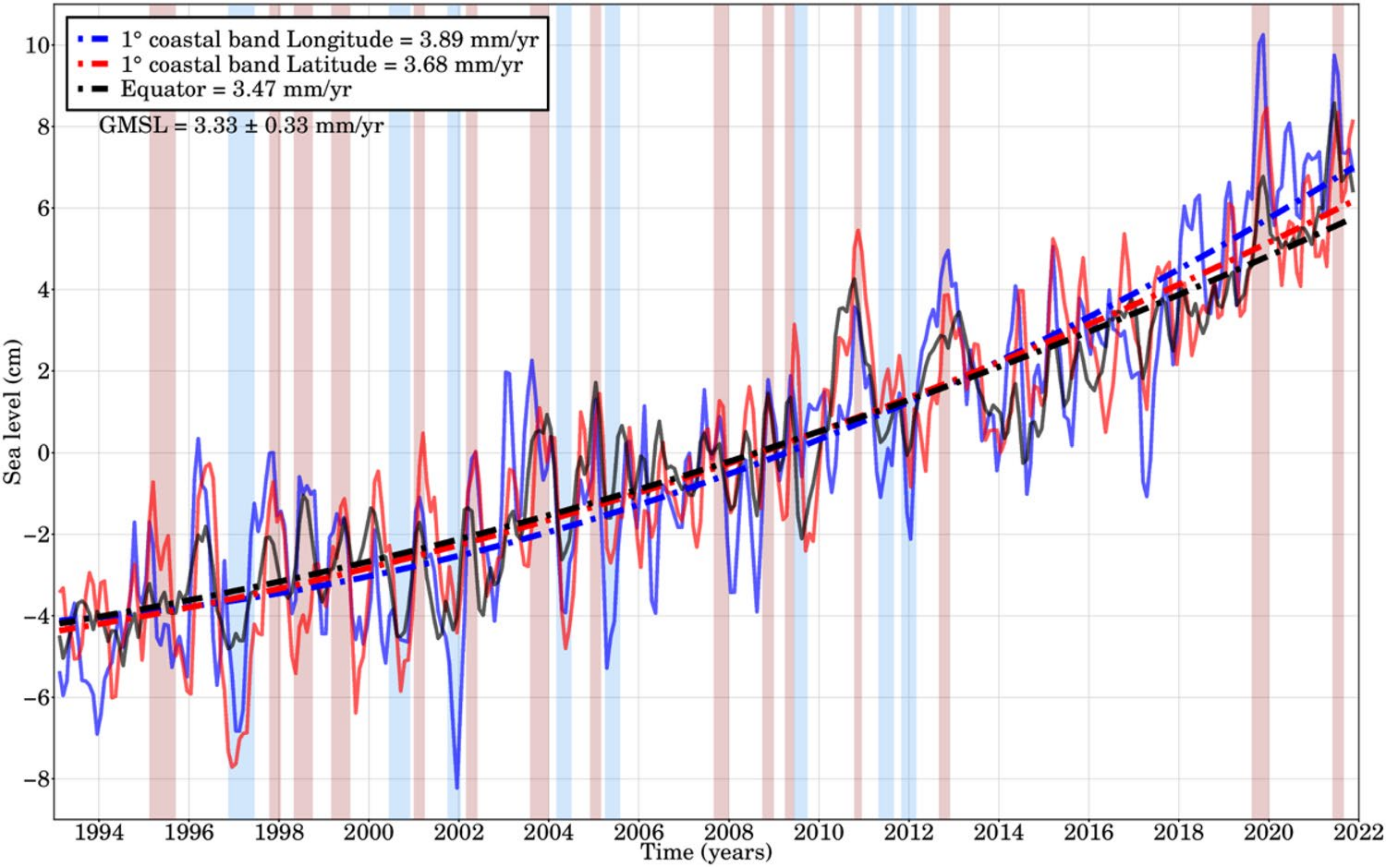
Altimetry-based sea level time series (cm) for the equatorial and GoG coasts from January 1993 to December 2021. Periodic terms (annual and semi-annual cycles) have been estimated and removed; glacial isostatic adjustment correction (ICE-5G)^50^ has been applied. Lines represent the quadratic trend associated with each domain. An acceleration of 0.094 ± 0.050 mm/yr^2^ was found which is almost similar from the GMSL acceleration estimated to 0.1 mm/yr^2^ from Cazenave et al.^47^ and Guérou et al^42^. The red and blue rectangles highlight the 8 Benguela Niñas (light blue) and 15 Niños (light pink) occurrences and where the width is a function of the duration of each episode.

Along the Angola-Benguela front, where the poleward-moving warm Angola Current and the northwest offshore-drifting cold Benguela Current converge, the warming rates are higher^45^. To investigate the impact of rapid warming trends on the sea level temporal evolution in the GoG, the analysis focused on the annual mean SLAs. This exploration within the dynamic framework of the tropical Atlantic's oceanic dynamics reveals thermal expansion as a pivotal link between SST and SLAs. The tropics, particularly the Atlantic region, showcase heightened sensitivity to temperature shifts, where water temperature profoundly influences seawater volume. Notably, the SST in this area has increased by up to 0.04 °C per decade, which has been associated with significant ecosystem changes^46^. According to Sweijd and Smit^39^, the GoG experiences the most rapid warming trends compared to the Angola-Benguela frontal zone, where cooling occurs at the coast. The annual mean SLAs were calculated by averaging the daily SLAs and were subsequently used for trend estimation (see Methods section). The rise in SST triggers two primary mechanisms that contribute significantly to the observed positive SLAs prevalent in the region. First, the warming of the upper ocean, driven by heat fluxes and potential changes in the freshwater content of the upper ocean, causes thermal expansion. At the same time, dynamically forced thermocline shoaling, coupled with thermocline feedback, increases SST. It's important to note that while thermocline depth is typically strongly correlated with SLA^20^, this relationship does not always hold on longer time scales. This intricate interplay of processes highlights the complexity of the ocean dynamics affecting both SST and SLA in the region. The time series in Fig. 3 shows the sea level temporal evolution of signals and trends in the equatorially driven zone and along 1° off the coasts of the GoG from January 1993 to December 2021 as shown in Fig. 1a. The sea level time series have been quadratically detrended to emphasise the interannual variability. Coastal sea levels show significant regional interannual variability (> 8 cm) driven by local coastal processes and Atlantic Niño events, as seen in the late 2019 and summer 2021 events, whose amplitude is reported to increase progressively poleward during these typical warm anomalies along coastal areas (see Fig. 3). The rate of sea level rise along both coasts of the GoG (3.68 to 3.89 ± 0.10 mm/yr) is slightly higher than that along the equatorial band (3.47 ± 0.10 mm/yr), while also higher than the global mean sea level trend (3.33 ± 0.33 mm/yr^42^). The mean trend difference between the coastal and equatorial bands is ~ 0.42 mm/yr. Since 2012, sea level change in the GoG has exceeded the equatorial variability. This has led to an acceleration in the region of 0.094 ± 0.050 mm/yr^2^ which is almost similar to the GMSL acceleration estimated to 0.1 mm/yr^2^ from Cazenave et al.^47^ and Guérou et al.^42^ resulting from the accelerated melting of Greenland and Antarctica ice sheets. On the other hand, this outcome, as described by Prigent et al.^48^; Worou et al.^49^, is consistent with the gradual decrease in Equatorial Atlantic SST anomalies associated with the reduction of the Atlantic Niño along the equatorial domain since 2000, resulting in the eastern Equatorial Atlantic thermocline being deeper and less responsive to Atlantic Niño events.

### Propagation of SST and SLA anomalies in the Gulf of Guinea in Late 2019 Benguela Niño events.

Sea surface temperatures in the Equatorial Atlantic are characterised by interannual variability which indirectly exerts an influence on sea level and precipitation over the surrounding continents, notably over the GoG through the West African monsoon dynamics. Although not immediately evident, these SST fluctuations indirectly affect seawater density (steric effect) and volume, consequently influencing the sea level. This relationship directly correlates SST variability with SLA in tropical regions, a connection primarily driven by the thermocline feedback mechanism. Changes in sea level may be associated with changes in the depth of the thermocline, potentially influencing adjustments in SST over time. In some cases, a decrease in sea level may lead to an upward movement induced by upwelling, and this association could contribute to a delayed decrease in SST. This intricate relationship underscores Equatorial Atlantic SST fluctuations' influence on regional climate patterns, particularly within the GoG.

**Figure 4:**
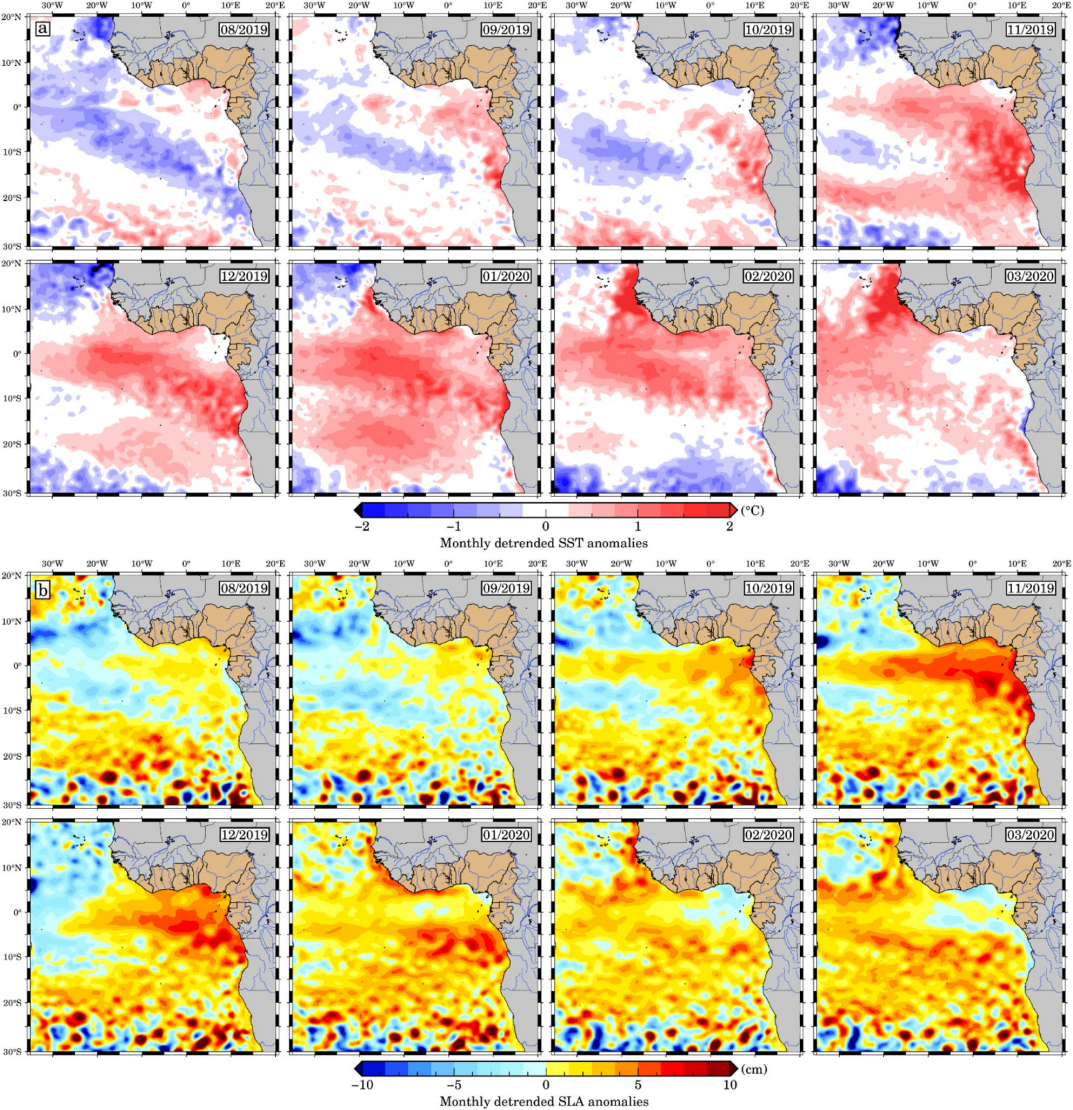
Monthly detrended (**a**) SST anomalies and (**b**) SLA averaged from August 2019 to March 2020. Burlywood colours represent countries of the GoG and blue lines across Africa indicate major river networks. The maps have been created using Generic Mapping Tools (GMT), Version 6.5.0 (https://www.generic-mapping-tools.org/).

Furthermore, the Equatorial Kelvin waves, induced either by zonal winds at the equator or through the reflection of equatorial or off-equatorial Rossby waves, propagate as CTW along the coast after reflecting at the western boundary. These signals can be measured by satellites, and their sea level signature provides sufficient information to identify them^19,51–54^. Over the past 20 years, interannual variability in ocean surface temperatures in the eastern equatorial Atlantic has been relatively low. However, in late 2019 (Fig. 4a) we witnessed an unexpectedly strong event that was reported to be the warmest of the last four decades which has been spread over the entire Tropical Atlantic^20,36^. Throughout the southeast Tropical Atlantic and along the Canary Current, negative SST anomalies prevailed during August 2019 at the start of the Benguela Niño event, with weak warming and positive SLA (4 cm, Fig. 4b) in the GoG and north of ~ 15°S. In September 2019, a month later, warming is observed further west of the GoG and along the coast north of 20°S with SST anomalies above 1 °C. The warming continued to spread south of 20°S in October 2019 in the Northern Namibia regions while the SLA maps displays the initiation of a positive signal covering almost ETA, as well as off Southern Angola and the Angola Benguela front regions. November 2019 marks the apex of the 2019 Benguela Niño, with high SST anomalies exceeding 2 °C in the Angola-Benguela front region and we can observe a signal propagation likely more identifiable as Kelvin coastal trapped waves which are found to spread in the GoG^19^. The entire GoG and southeast Atlantic are abnormally warm at the event's climax. However, it's important to note that CTWs are not the only contributors to this warmth. Notable differences between SST and SLA signals along the GoG coast, especially in November, reveal a complex interplay. Low SST near the Niger delta plumes coincides with high SLA. River discharges, especially from the Niger delta plume, introduce freshwater into the GoG, which influences the SST through dynamic interactions with seawater^28^. At the same time, the shape of the coastline and regional bathymetry, which are critical for CTW propagation, play a key role in modifying the dynamics of temperature anomalies along the coast. Changes in river dynamics and coastline morphology can alter the interplay between CTWs and other oceanic processes^37^, further influencing the observed SST/SLA dynamics. These patterns persist across regions and months, highlighting the critical role of local processes, including CTWs, in shaping the intricate temperature anomalies observed in the GoG and the southeast Atlantic.

The widening of the band of positive SLA anomalies (> 3 cm) at the equator is observed 30 days after the peak of the equatorial Kelvin wave (Fig. 4), typical for reflected westward propagation of equatorial Rossby waves^55^. CTWs produce signals in the SLA that are often associated with downwelling (or upwelling) correlated with sea level rise (or fall). Poleward propagating CTWs not only affect near-coastal sea level, but they are also connected with distinct spatial patterns of velocity and density anomalies that vary depending on stratification and the local topography of the shelf and continental slope^28^. The onset of cold surface waters in the northern Namibia (southern Angola) and Angola-Benguela front regions in January/February 2020, signals the end of the anomalously warm event, with contrasting warm surface waters and positive SLA anomalies in the GoG showing propagation during these 2 months until March. SLA and SST anomalies in both GoG and southern Angola reach their maximum in January 2020, but only near the coast. In March 2020, the GoG region showed negative SLA and cold SST anomalies extending westwards to Côte d'Ivoire and northwards along the coast of southern Angola. Richter et al.^20^ highlighted that, apart from dry anomalies over the coastal region of equatorial Africa, precipitation anomalies over land were very inconsistent during the event. Polo et al.^19^ assessed the propagation of Kelvin waves with periods between 25 and 95 days along the equatorial and coastal wave tracks, which is consistent with the late 2019 event.

### Kelvin wave propagation from PIRATA moorings and Argo steric sea level data

The redistribution of heat by the ocean circulation, influences the spatial patterns of temperature change in the ocean, thereby impacting steric sea level and contributing to sea level anomalies. The increase in temperatures leads to a drop in seawater density, resulting in thermal expansion (thermosteric) and playing a substantial role in steric sea level fluctuations.

**Figure 5:**
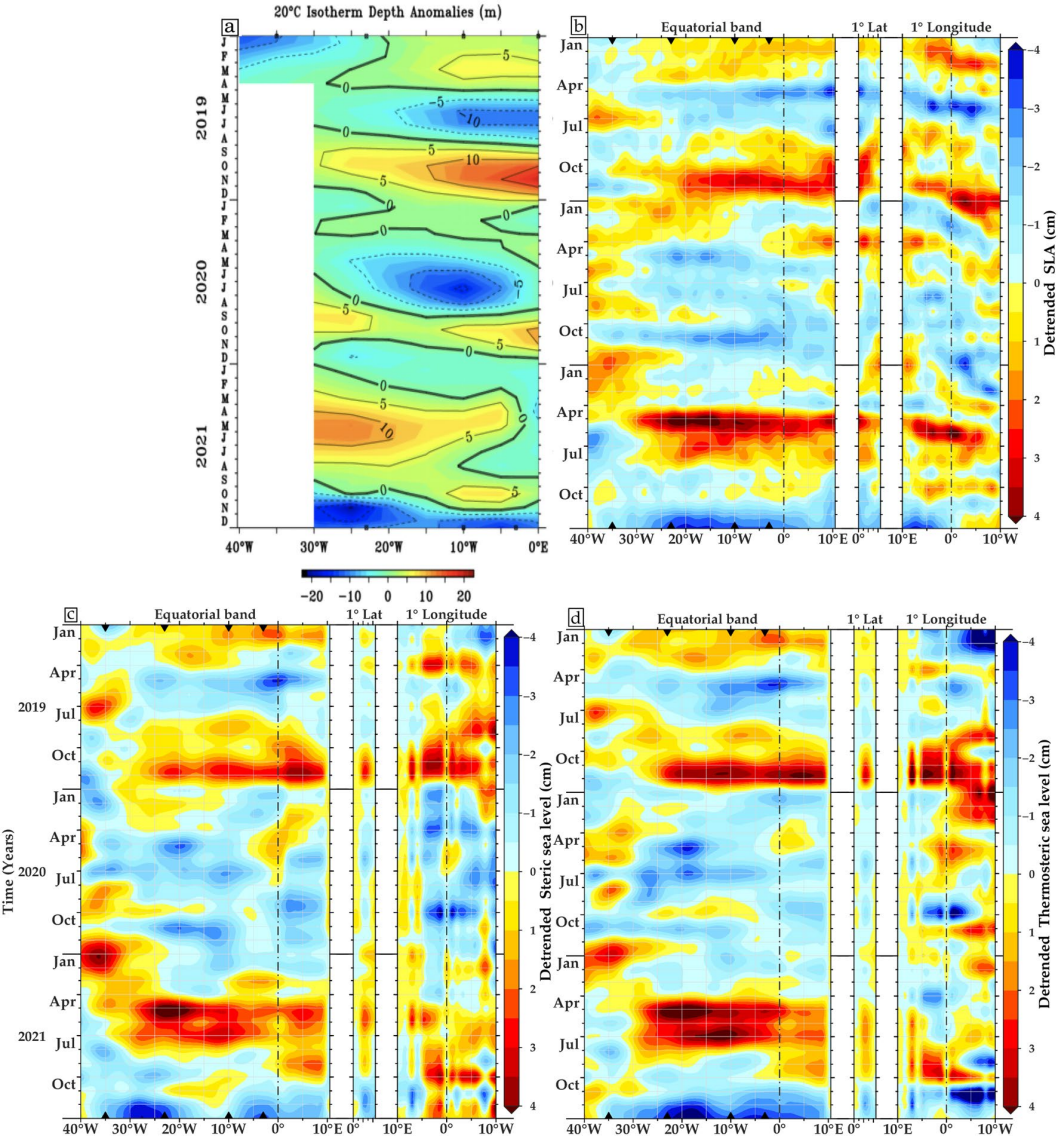
Hovmöller diagrams of (**a**) monthly mean of the D20 anomalies (m) along the equator, inferred from the PIRATA moorings, (**b**) detrended monthly SLA, (**c**) detrended monthly steric sea level (0–700 m) and (**d**) detrended monthly thermosteric sea level (0–700 m) from January 2019 to December 2021. The equator (averaged over 1°S–1°N) from 40°W to the African coast, and the GoG coast (averaged from coast to 1° offshore, from 0°N to 4.5°N, as well as between 10°E and 10°W). The calculation of anomalies considers the period from January 1993 to December 2021 (Note that the y-axis has been reversed to match PIRATA maps). Dots and triangles observed on the upper and lower x-axis along the equatorial band represent four active PIRATA moorings. The steric and thermosteric sea level components are based on the gridded ensemble mean of the Argo float sources (details in the Methodology section). The maps have been created using Generic Mapping Tools (GMT), Version 6.5.0 (https://www.generic-mapping-tools.org/).

The significance of the variations in depth within the D20 which in general is located at average depth of 140 m in the western and 50 m in the eastern Atlantic on the equator^21^ lies in their relevance to understanding the link between temperature and density, which is essential to understand the steric sea level changes. Figure 5 shows the D20, detrended sea level, Argo-based steric and thermosteric anomalies from the ensemble mean of four Argo float data sources between January 2019 and December 2021 along the Equatorial Atlantic and the GoG domains (shown in Fig. 1).

The equatorial wave propagation along the equatorial and coastal waveguides is well observed using SLA altimetry data. Figure 5a clearly shows the pattern of eastward propagation of the PIRATA records. This allows us to estimate the time lags at different longitudes for some specific strong eastward propagations, and to compare the projected phase velocity values with the velocity of each baroclinic mode.

We focused on the PIRATA D20 interannual anomalies because changes in the thermocline depth of less than 20 m (deepening or shoaling) are a key aspect of the Interannual Equatorial Kelvin Waves (IEKW).

As seen in Fig. 5 a positive anomaly of SLA is visible in the Equatorial Atlantic from October to December 2019, which is consistent with the 20 °C isotherm depth from the PIRATA data (Fig. 5a). However, the SLA signal indicating a downwelling EKW is rather moderate compared to other Benguela Niño events, such as the one that occurred in 2010/2011^56^. Also, the D20 from the PIRATA moorings in October and November 2019 and April–July 2021 show an equatorial thermocline that is deeper than usual (> 15 m) due to the activity of the downwelling EKW. However, positive anomalies of the 20 °C isotherm depth and SLA show an eastward propagation from 10°W towards the African coast and along the northern coast of the GoG, illustrating the poleward propagation of this signal, well captured by both steric and thermosteric sea level anomalies as a coastal trapped Kelvin Wave both in late 2019 and in boreal summer 2021. Notably, within the GoG coastal regions, the Argo-based steric and thermosteric sea level data (Fig. 5c,d) serve as valuable indicators reflecting the propagation of CTWs. In agreement with the studies of Illig et al.^57,58^; Jouanno et al.^59^, this propagation is indeed congruent with a spontaneously propagating between the first and second baroclinic mode downwelling Kelvin wave with a phase velocity between 1.4 and 2.5 m/s.

### Interannual sea level variability during the altimetry era (since 1993)

**Figure 6:**
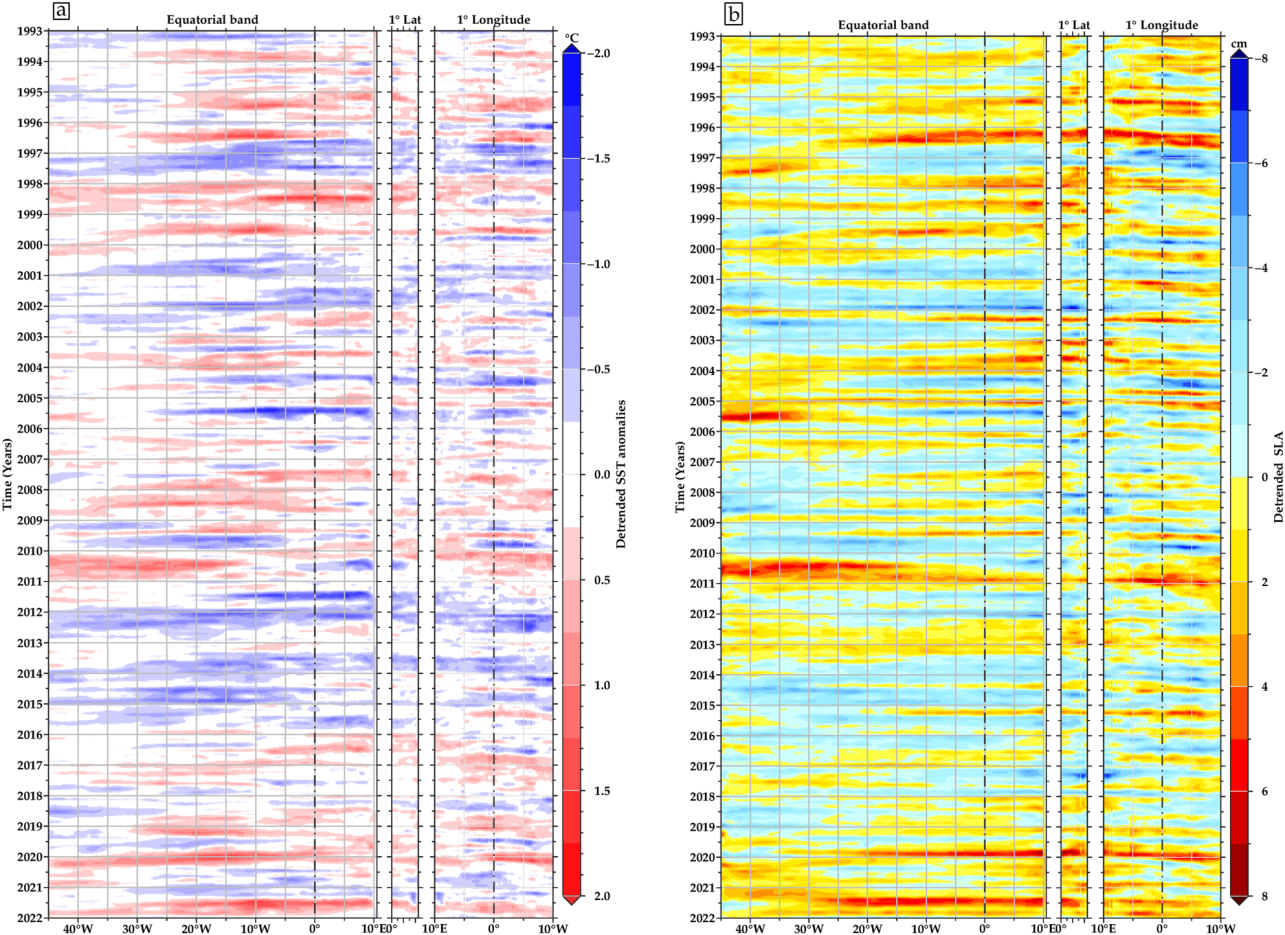
Hovmöller diagrams exhibiting monthly detrended (**a**) SST and (**b**) SLA in longitude-time (Equatorial domain), latitude-time (1° coastal band latitude), and longitude-time (1° coastal band longitude). Along the GoG coast domains are averaged from the shoreline to 1° offshore. The size of the diagrams is proportional to the selected areas, which range from the open sea to the coasts, as shown in Fig. 1a. The maps have been created using Generic Mapping Tools (GMT), Version 6.5.0 (https://www.generic-mapping-tools.org/).

**Figure 7:**
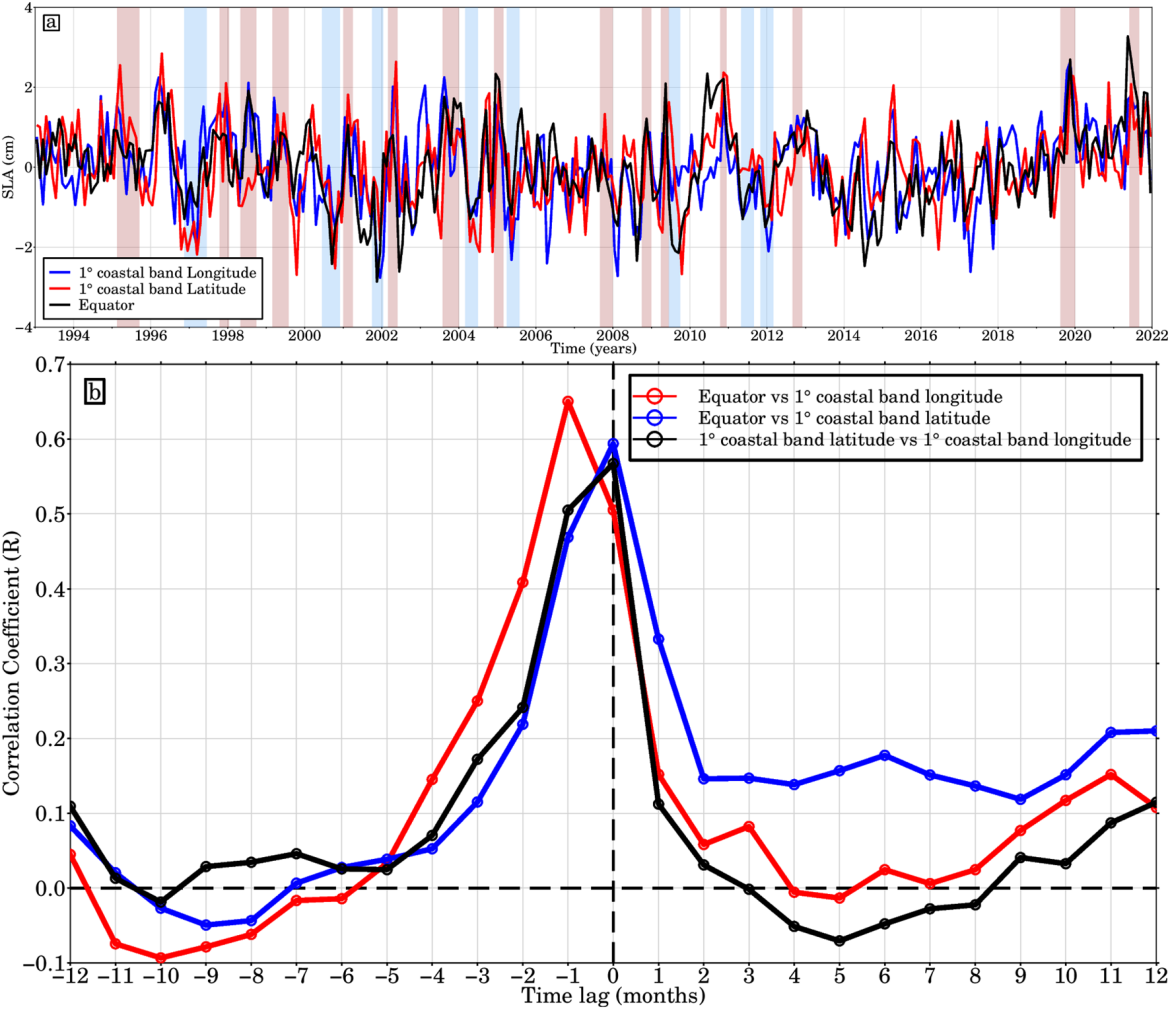
(**a**) Normalised interannual detrended SLA monthly averaged in the equatorial domain and the GoG (over 1° coastal area along latitude and longitude) between January 1993 and December 2021. The red and blue rectangles highlight the 8 Benguela Niñas (light blue) and 15 Niños (light pink) occurrences and where the width is a function of the duration of each episode. (**b**) Cross-correlation of monthly sea level anomalies (SLA) averaged over the equatorial domain (50°W–17°E; 3°S–3°N) and along the GoG, encompassing both the 1° coastal latitude area (5°E–15°E; 0°N–4.5°N) and the 1° coastal longitude area (10°W–10°E; 3°N–9°N), as a function of lag (in months).

Imbol-Koungue et al.^61^ found multiple notable upwelling and downwelling of IEKW, primarily associated with the episodic Benguela Niño (related to abnormal downwelling IEKW SLA positive signatures) and Benguela Niña (abnormal upwelling IEKW SLA negative signatures) events, while in the GoG non-existing related events have been reported. According to their coastal SST criterion, 23 events were identified and classified. Eight cold events took place from the 1996–2012 period, while fifteen major warm events occurred from 1995 to 2021 period (see Fig. 7a). All these exceptional events have already been discussed in the literature^20,60–68^. Figure 6 depicts most of the negative and positive anomalous propagation episodes, including the late 2019 (see Fig. 4) and boreal summer 2021 events. The results confirm the propagation of SLA and SST anomalies and reveal the connection between the equatorial region and GoG coast. Notably, SLA shows increased accuracy compared to the SST in delineating this connection, particularly during warm events noted in 1996, 1999, 2002, 2010, 2019, and 2021. This preference for SLA’s precision could be attributed to its close correlation with the thermocline. The thermocline's influence on SLA, mirroring shifts in water masses and density fluctuations, underscores SLA's effectiveness in depicting anomalies of oceanic currents and circulation patterns, and broader climatic variations compared to SST. The equatorial Kelvin Waves are often well documented in sea level and D20 anomalies because they are connected to a shift in the thermocline and SLA. A 3-m deepening of the thermocline is roughly equivalent to a 1-cm rise in SLA^69^. According to Philander et al.^70^, thermocline shifts are proportional to SST anomalies, with downward/upward shifts corresponding to warm/cold SST anomalies, respectively. This relationship is indicative of a thermocline feedback mechanism as discussed by Imbol-Koungue and Brandt^71^, wherein vertical mixing acts to cool or warm the sea surface during upwelling or downwelling events, respectively. They further elucidate that this thermocline feedback exhibits a delayed response, typically manifesting over a period of about 14 days. Vertical shifts of the thermocline have a significant impact on SLA variations in a hydrostatic equilibrium^72^.

Detrended SST and SLA anomalies (Fig. 6) along the coast of the GoG and the Equatorial Atlantic are helpful for illustrating the spread of downwelling EKWs and subsequent CTWs. This indicates that the IEKW and SLA episodes propagation identified by the current study are also spreading as CTWs, throughout the GoG coast, highlighting the influence of both remote and local wind forcing. In particular, local longshore wind-forced CTWs play a significant role in enhancing the SLA signal, especially when they coincide with increased coastal wind activity^28^. In addition, the boreal summer emergence of a strong Benguela Niño in April 2021, persisting through this period and concurrently manifesting as extreme warm events in the GoG, can be attributed to CTWs (as shown in Fig. 6), reported by Crespo et al.^68^ to be larger than the late 2019 event. These findings underscore the substantial role played by CTWs in regional climate dynamics, aligning with observations of the GoG warming in 2010 and experiencing drastic cooling in 2012, as previously reported by Da‐Allada et al.^17^.

Moreover, the onset of the severe cooling in 2012 (February–June) did not coincide with the June-July–August period typically characterised by the Atlantic Niño (or equatorial) mode. Instead, it occurred during the peak of the Benguela Niña. Furthermore, Awo et al.^73^ found that there was no Atlantic Niño in GoG in 2012. These results imply that the unusually cold SST event that occurred in February–June 2012 cannot be attributed to the equatorial variability in GoG^17^ but was primarily caused by eastward transport and enhanced turbulent mixing near Cape Palmas due to a strengthening Guinea Current and vertical shear. In the eastern region near Cape Three Point, where seasonal upwelling is mostly wind-driven, the event was caused by more eastward wind stress, which boosted Ekman transport and offshore water movement^21^. The 1996 warm event followed by a cold event^25,74,75,78^ in the eastern equatorial part is well marked in the amplitude of the interannual variability in the equatorial part as well as in two GoG coastal areas (see Fig. 1a). In this region, a significant correlation (r = 0.68) of the SLAs between the equatorial domain and the coastal domain along longitude is observed, with the anomalies following the same trends throughout the period with a lag correlation showing up to 1 month signal propagation to GoG (Fig. 7b). Most of the SLA anomalies correspond to warm and cold events that have been recorded in southern Africa over the ocean and have been linked to IEKW upwelling and downwelling propagations, respectively. In addition, the occurrence of these warm events is preferentially in the boreal summer during the months of MJJA with amplitudes greater than 0.5 °C (Fig. 6), which is above the climatological average. Consistent with the dynamics of Atlantic Niño events^76^, the passage of the equatorial Kelvin Wave, identified by its sea level signature, is followed by a warming of the SST. An interesting temporal synchronisation between the eastward propagating equatorial Kelvin Waves and the reflection along the northern tropical Atlantic coast (GoG) is revealed by positive or negative SLA. The results show that IEKW events detected in Benguela coincide with the detrended SLA events along GoG coast between 1993 and 2021. However, it is noted that several events do not correspond to boreal summer occurrences^28^. Furthermore, specific IEKW propagation events, such as the 1998, 2001/2002 and 2009 events associated with Rossby wave reflections as noted by Imbol-Koungue et al.^60^, show good agreement with coastal SLA events, indicating the influence of IEKWs rather than the amplitude of the tropical western Atlantic wind stress.

## Discussion and conclusions

The study aims to present observation-based insights into the propagation of CTWs towards the GoG coast. It delves into three primary aspects: (1) investigating trend variability of the SLAs in the Tropical Atlantic, especially the GoG, (2) analyzing the equatorial Kelvin waves and CTW propagation from the Equatorial Atlantic to West Africa coast through SST and SLA, and (3) examining PIRATA moorings and Argo-based steric and thermometric sea level data from 1993 to 2021. The oceanic processes associated with the generation of sea level variations range from interannual to decadal timescales associated with remote forcing such as coastal trapped wave dynamics, tides and local vertical land motion. Using both altimetric monthly SLAs and SST anomalies, we found that the GoG has experienced four warm events over the past 10 years, with the most recent two, which were exceptionally strong (warmest during the satellite altimeter era) and occurred in late 2019 and boreal summer 2021 (Fig. 7). Illig and Bacherely^37^ observed a long-term oceanic memory resulting from free-propagating downwelling equatorial waves forced before 2021. These waves warmed the slightly colder than normal surface layer near the equatorial waveguide during the first trimester of 2021, leading to neutral conditions by March 2021, just before the onset of an equatorial event. This facilitated a positive feedback loop between positive SSTAs in the GoG. Lee et al.^38^ investigated this phenomenon further, revealing the generation of three downwelling Kelvin waves as Rossby waves reflected off the South American coast during the winter and spring of 2021. Two of these Kelvin waves, which occurred in early to mid-January and mid to late March, propagated eastward along the equatorial Atlantic. Another reflected Kelvin wave, generated in mid to late April, developed into a full-scale downwelling Kelvin wave and reached the coast of the GoG, attributed to a Madden–Julian Oscillation-driven wind burst event in the first week of May. In line with the causal relationship between warm eastern equatorial Atlantic SSTAs and an enhanced Atlantic Intertropical Convergence Zone rain band, heavy rainfall and several flooding events^77^ were reported in the GoG. It has been shown that coastal downwelling Kelvin waves has deepened the thermocline in the east along the equatorial region of the Equatorial Atlantic (see Fig. S1 Supplementary material). These waves have also effectively propagated northwards along the GoG as coastal Kelvin trapped waves at 1.4 m/s, coincident with the onset of the Benguela Niño and currently GoG Niño events. As a result of the GoG's extensive stratification, the CTWs, which are largely dependent on the cross-shore topography and stratification^58^, exhibit properties that are comparable to those of coastal Kelvin waves^78^. In this regime, the CTWs are at first order comparable to coastal Kelvin waves that follow a sloping coast. In addition, they also show that the strength of the coastal trapped signal is greater in the southern hemisphere compared to the northern hemisphere, based on the utilization of a realistic shoreline. Interannual SLA along the equatorial domain are systematically examined, and it is discovered that they advance the West African coastal ones by 1 month. According to Richter et al.^20^, the primary cause of the warm SST anomalies were wind stress curl anomalies and westerly wind anomalies north of the equator (along West Africa). Increased heat storage from recurrent warm events will increase water temperature, leading to thermal expansion of seawater in the GoG. Therefore, assessment of ocean heat storage will be an important factor in addressing the regional sea level rise due to recurring warm events. The D20 observation from PIRATA moorings offer essential insights into the oceanic conditions within the Equatorial Atlantic. However, the sparse distribution of these moorings (only 4 active moorings along the equatorial band), particularly limited in the open ocean, restricts their effectiveness in capturing localized variability, especially in coastal regions such as the GoG. In contrast, Argo-based steric and thermosteric sea level data provide a more comprehensive and spatially representative understanding of sea level variations, extending from the equatorial domain to the GoG coastal areas. It is worth noting that Argo floats highlight the challenge of accurately capturing coastal dynamics, particularly in shallow waters, such as the poleward propagation of both steric and thermosteric sea level anomalies as coastal trapped Kelvin waves. Our analyses overcome this limitation by assimilating SLA data from satellite altimetry and other sources, reanalysis data provide insights into past events and long-term trends. The results effectively bridge the gap between observational datasets and numerical models. This integration significantly improves our understanding of coastal wave dynamics and their impact on coastal oceanography. Our analysis also highlights the significant impact of climate change on ocean heat storage, which plays a crucial role in driving sea level variability. Increased ocean heat uptake enhances thermosteric sea level fluctuations, resulting in the observed anomalies in the coastal regions of the GoG. Furthermore, in the tropical Atlantic, increased heat storage enhances thermosteric sea level fluctuations, contributing to the observed sea level variability during recent extreme events. These fluctuations directly affect coastal communities in the GoG by increasing the frequency of coastal flooding and erosion. The relationship between ocean heat content and sea level anomalies highlights the importance of understanding and monitoring these phenomena for effective coastal management and adaptation strategies. By gaining insight into the underlying mechanisms driving sea level variability, coastal communities can better prepare for and mitigate the impacts of sea level rise in the GoG.

Observations from PIRATA moorings, while valuable for understanding general thermocline variations along the equatorial band, lack the spatial coverage required to accurately depict coastal phenomena such as CTWs. Conversely, Argo floats, with their broader distribution and coverage closer to coastal regions, offer more reliable in-situ measurements for capturing CTW propagation and related steric and thermosteric sea level variations in the GoG.

## Methods

The gridded daily SLAs at 1/4-degree spatial resolution from January 1993 to December 2021 from the delayed-time multi-mission (all satellites merged) and the near-real-time datasets distributed by the European Union Copernicus Marine Environment Monitoring Service (CMEMS) Level 4 (L4) have been used in this study.

To investigate SST variations, we used the NOAA Daily Optimum Interpolation Sea Surface Temperature V 2.1 dataset, with a spatial resolution of 0.25° × 0.25°, covering the period 1993–2021^79^. The data are derived from daily merged in situ and remotely sensed data.

**Figure 8:**
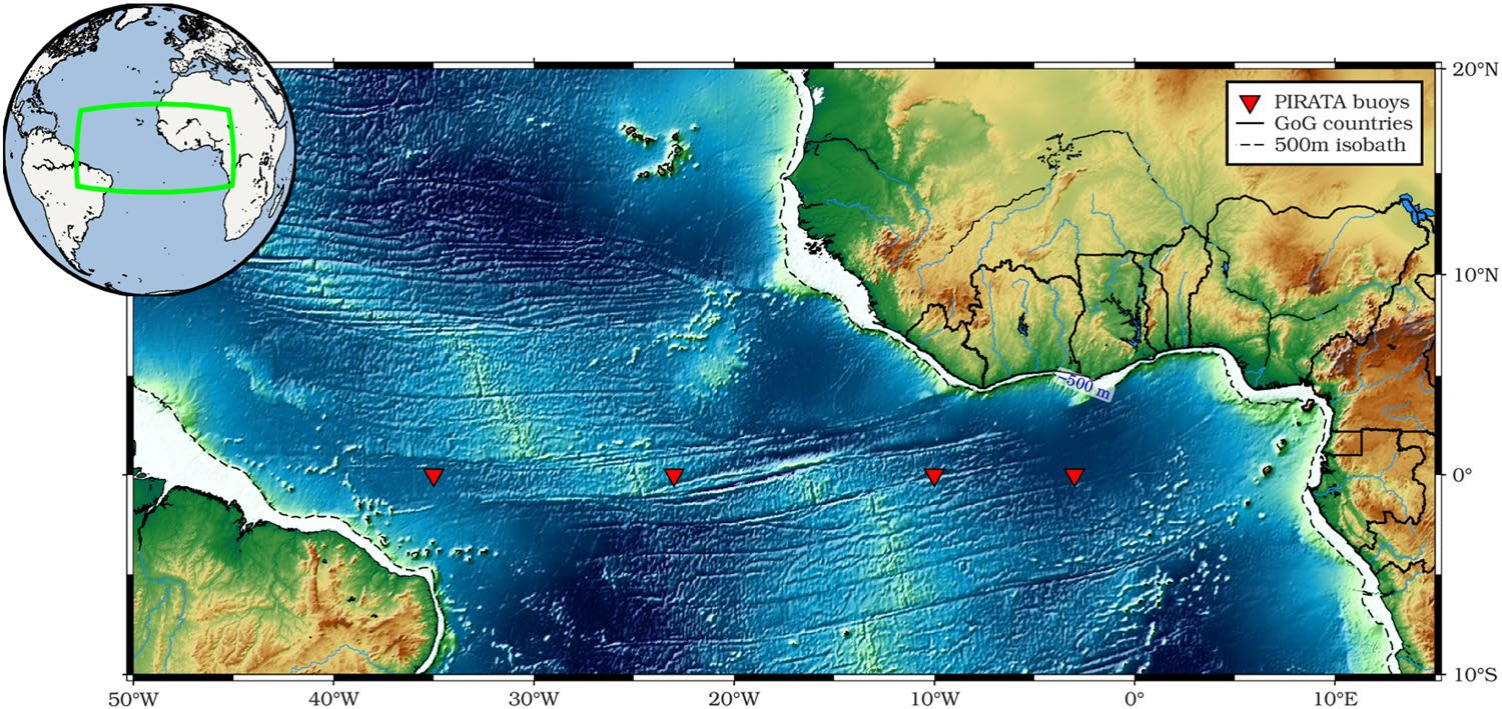
Map of study area showing the location of the four PIRATA buoys used. The map has been created using Generic Mapping Tools (GMT), Version 6.5.0 (https://www.generic-mapping-tools.org/).

In order to derive trends that accurately reflect the region's net sea level rise, we applied the Glacial Isostatic Adjustment^50,80^ (GIA) correction (of about − 0.3 mm/yr in the ETA, computed using the ICE5G-VM2 model downloaded from https://www.atmosp.physics.utoronto.ca/%7Epeltier/data.php). Figure 2b shows a rise in sea level everywhere in the ETA Ocean during the last three decades spanning from 2 to 6 mm/yr. Sea level change rates along the GoG coastline are higher than in the remote Tropical Atlantic. The average trend in the Tropical Atlantic Ocean is 3.57 ± 0.10 mm/yr slightly higher rate than the global mean sea level (GMSL) trend of 3.33 ± 0.33 mm/yr (at the 90% confidence level) during 1993–2021^41,42,81^. Based on Ablain et al.^82^, who quantified the sources of errors impacting all components of the altimetry system (drifts, biases, and noises), the estimate of the GMSL has an uncertainty of 0.3 mm/yr. As one gets closer to the coast, the trends in the coastal regions of the GoG and Angola become noticeably larger (over 4.3 mm/yr) which is 25% higher than the GMSL during 1993–2021. Regional sea level changes in the Tropical Atlantic, in contrast to the global average, are significantly biased by internal modes of climate variability that occur on interannual to multi-decadal timescales, which mask any long-term change (i.e., low signal-to-noise ratio), including: atmospheric loading; local/regional changes in sea water density due to temperature and salinity changes (steric effects); the ocean circulation's redistribution of ocean water mass (known as the manometric component); and the solid Earth's deformations and gravitational changes in response to mass redistributions^42,83,84^. Since 1997, PIRATA current-meter array records have been used in the Tropical Atlantic to detect and monitor the propagation of Kelvin and Rossby waves along the equator. The data, gridding procedure and climatology estimate can be found at: http://www.pmel.noaa.gov/tao/disdel/. Monthly dynamic height and anomalous depth of the thermocline (D20) from four PIRATA buoys at 35°W, 23°W, 10°W and 3°W (Fig. 8) were utilised to detect the EKWs propagation. At monthly intervals, we calculated an ensemble mean of 1° × 1° gridded steric data (0–700 m depth range for integrating salinity and temperature effects based on Guivarc’h et al.^78^) from four gridded ocean temperature and salinity datasets released by the following institutions and listed in Table 1: the Barnes objective analysis (BOA, ftp://data.argo.org.cn/pub/ARGO/BOA_Argo/) dataset; the Met Office Hadley Centre for Climate Change (EN4 series products, version 4.2.2, named EN4_g10, https://www.metoffice.gov.uk/hadobs/en4/), the Scripps Institution of Oceanography (SIO, https://sio-argo.ucsd.edu/pub/Global_Marine_Argo_Atlas/) and the Japan Agency for Marine-Earth Science and Technology (JAMSTEC, https://pubargo.jamstec.go.jp/argo_product/catalog/aqc/catalog.html) dataset. In addition to Argo float measurements, the EN4 dataset also includes the mechanical (MBT) and extendable (XBT) bathythermograph data. The sole source of data for the JAMSTEC dataset is Argo float salinity and temperature measurements.

**Table 1:** Summary of Argo floats data sets used to calculate steric and thermosteric sea level components.

Institute	Spatial resolution	Temporal resolution	Verical resolution
EN4	1° × 1°	Monthly	42 levels to 5350 m
BOA	1° × 1°	Monthly	58 levels to 1975 dbar
JAMSTEC	1° × 1°	Monthly	25 levels to 2000 dbar
SIO	1° × 1°	Monthly	58 levels to 1975 dbar

Steric sea-level change is computed by using the Argo products as^85^

1 $${SL}_{steric}=\frac{-1}{{\rho }_{0}}\cdot {\int }_{-h}^{0}\Delta \rho \cdot dz$$

where, ${\rho }_{0}$ is the mean density of seawater (1027 kg/m^3^), $z$ denotes depth, *h* is the reference depth, which is set to 700 m and $\Delta \rho$ is the density change as a function of temperature, salinity and pressure, which can be computed using the United Nations Educational, Scientific and Cultural Organization (UNESCO) standard Eqs. ^86^. Since the mean salinity is used in Eq. (1), any salinity effect on steric sea-level change is not considered here and we only focus on the thermosteric contribution. For each of these variables, we subtracted the monthly climatology (calculated for the period 2005 to 2021) from the initial monthly time series to remove the seasonal signal and obtain the interannual anomalies.

### Supplementary Information

Supplementary Figures.

## Data Availability

All data that support the findings of this study are included within the article (and any Supplementary files).
