# Supplementary material for: Sea level variability in Gulf of Guinea from satellite altimetry
Source: Sci Rep. 2024 Feb 27;14:4759. doi: 10.1038/s41598-024-55170-x (PMC10899594; doi:10.1038/s41598-024-55170-x)
Supplement: Supplementary file 1 — Supplementary Figures. [file 41598_2024_55170_MOESM1_ESM.docx]

**SUPPLEMENTARY MATERIALS**

**Figure. S1:** *Monthly detrended 20*°*C isotherm averaged from August 2019 to March 2020 obtained from ORAS5. Burlywood colours represent countries of the GoG.*
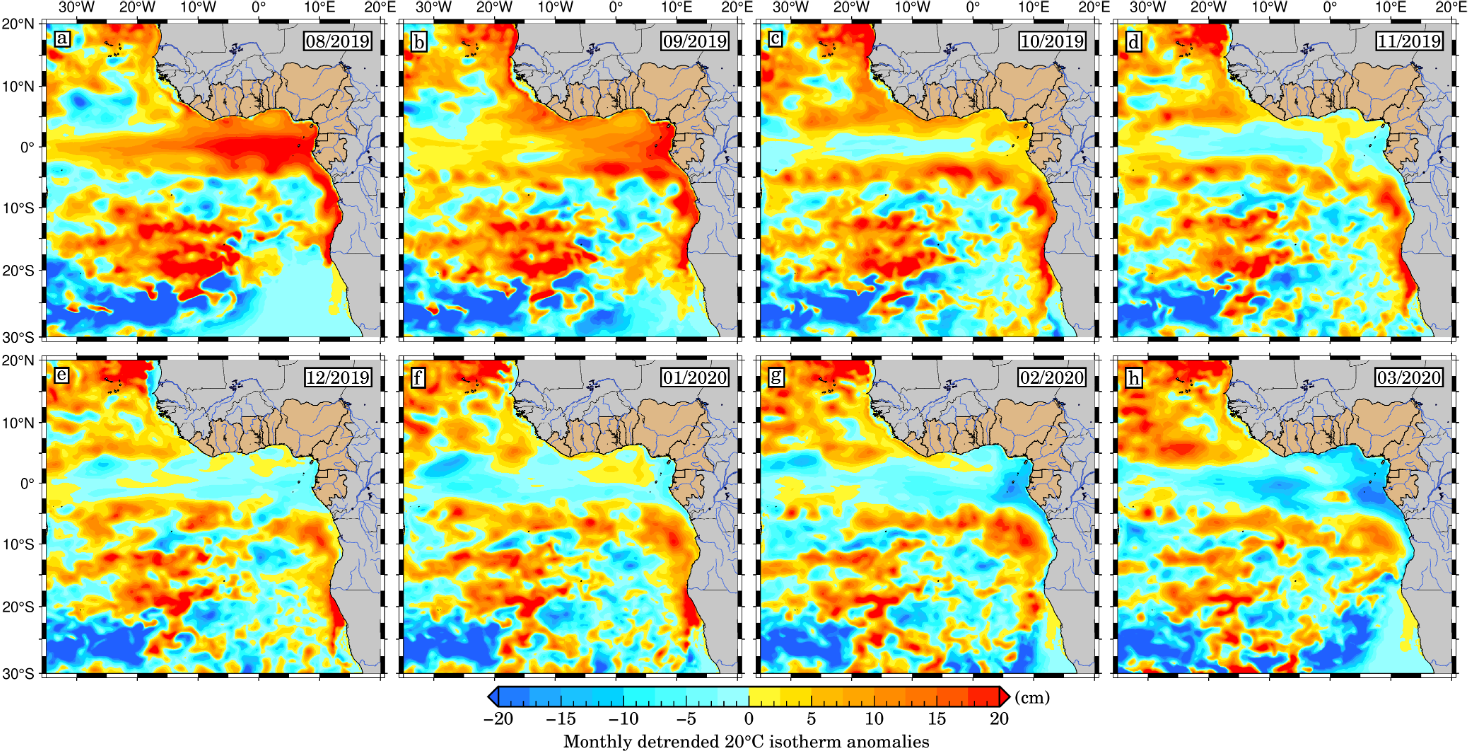


*.*


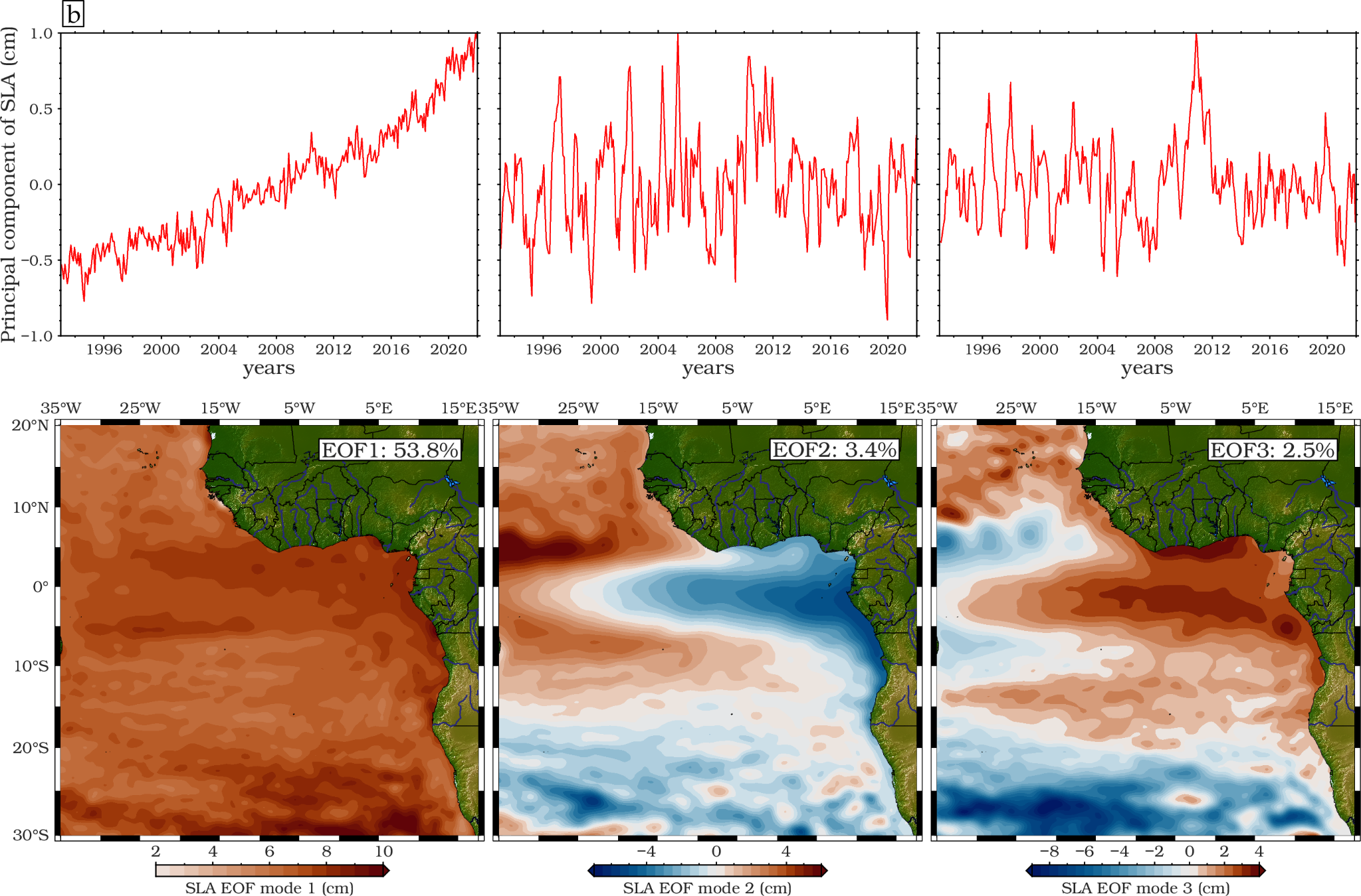

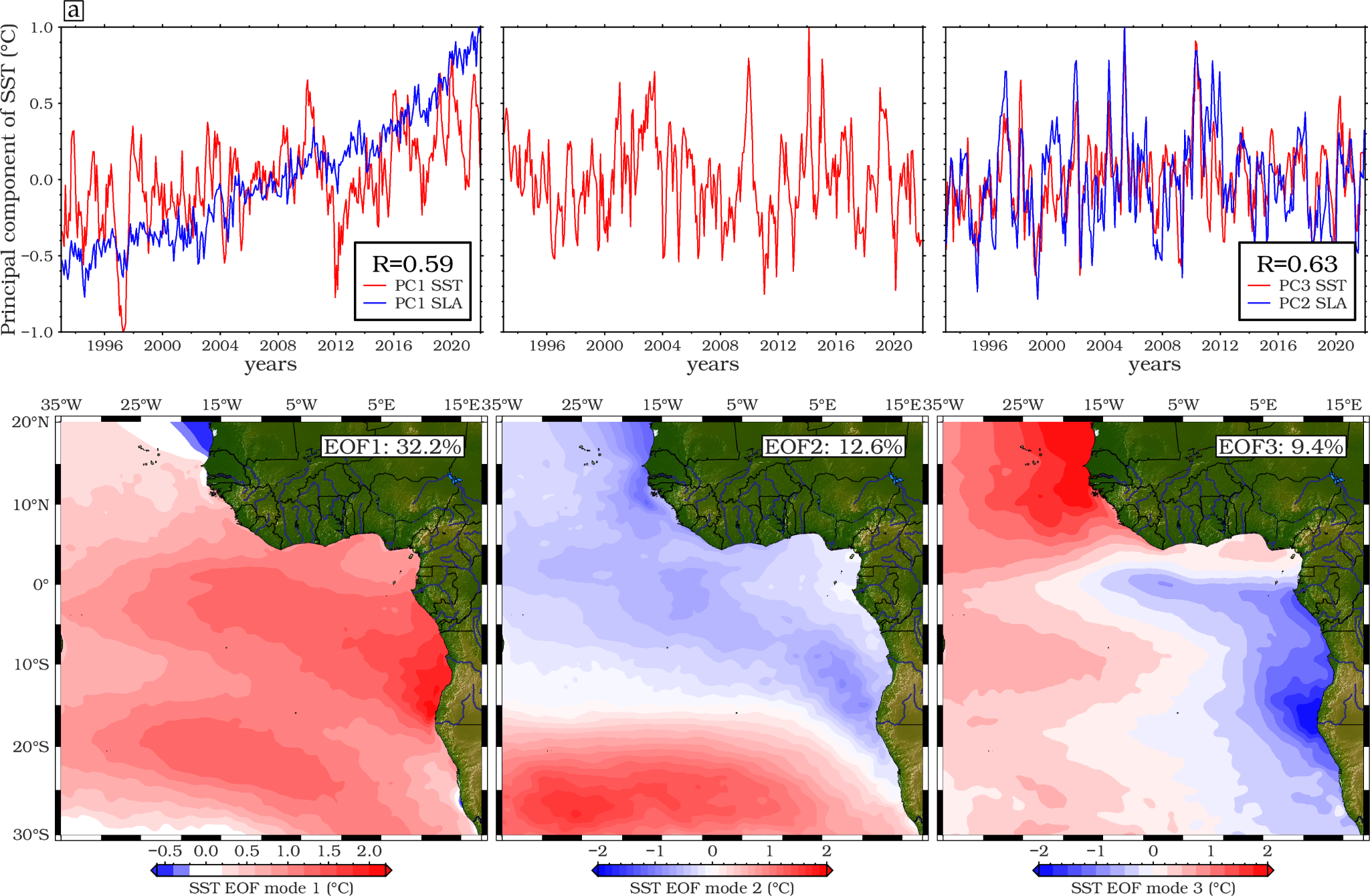
**Figure. S2:** *Modes 1, 2, and 3 of the empirical orthogonal function (EOF) decomposition of the gridded time series of the* ***a)*** *SST and* ***b)*** *SLA from 1993 to 2021. Top and bottom panels, respectively, display principal components and related spatial patterns. Each mode 1 shows a dominant trend signal, which is positive in the Eastern Tropical Atlantic. The results indicate a correlation coefficient of 0.59 between the mode 1 of the sea surface temperature (SST) and the sea level anomaly (SLA), as well as a coefficient of 0.63 between mode 2 of the SLA and mode 3 of the SST. The mode 1 of the SST and SLA presents a relevant representation of the trends associated with their respective variables.*
